# Rethinking non-inferiority: a practical trial design for optimising treatment duration

**DOI:** 10.1177/1740774518778027

**Published:** 2018-06-05

**Authors:** Matteo Quartagno, A Sarah Walker, James R Carpenter, Patrick PJ Phillips, Mahesh KB Parmar

**Affiliations:** 1 MRC Clinical Trials Unit at UCL, London, UK; 2 Department of Medical Statistics, London School of Hygiene & Tropical Medicine, London, UK

**Keywords:** Antimicrobial resistance, design, randomised trial, flexible modelling, non-inferiority, duration of therapy

## Abstract

**Background:**

Trials to identify the minimal effective treatment duration are needed in different therapeutic areas, including bacterial infections, tuberculosis and hepatitis C. However, standard non-inferiority designs have several limitations, including arbitrariness of non-inferiority margins, choice of research arms and very large sample sizes.

**Methods:**

We recast the problem of finding an appropriate non-inferior treatment duration in terms of modelling the entire duration–response curve within a pre-specified range. We propose a multi-arm randomised trial design, allocating patients to different treatment durations. We use fractional polynomials and spline-based methods to flexibly model the duration–response curve. We call this a ‘Durations design’. We compare different methods in terms of a scaled version of the area between true and estimated prediction curves. We evaluate sensitivity to key design parameters, including sample size, number and position of arms.

**Results:**

A total sample size of ~ 500 patients divided into a moderate number of equidistant arms (5–7) is sufficient to estimate the duration–response curve within a 5% error margin in 95% of the simulations. Fractional polynomials provide similar or better results than spline-based methods in most scenarios.

**Conclusion:**

Our proposed practical randomised trial ‘Durations design’ shows promising performance in the estimation of the duration–response curve; subject to a pending careful investigation of its inferential properties, it provides a potential alternative to standard non-inferiority designs, avoiding many of their limitations, and yet being fairly robust to different possible duration–response curves. The trial outcome is the whole duration–response curve, which may be used by clinicians and policymakers to make informed decisions, facilitating a move away from a forced binary hypothesis testing paradigm.

## Introduction

While much early-phase drug development focusses on identifying the most appropriate dose, for many conditions, less emphasis is placed on identifying the most appropriate treatment duration. Consequently, duration is often based as much on precedent as evidence. A motivating example is bacterial infections, where concerns about under-treatment and low costs have historically led to long antibiotic courses. However, widespread antibiotic overuse over the past decades, for example, for non-bacterial infections or for longer than necessary to cure an infection, is now considered the main driver for increasing antimicrobial resistance.^1,2^

How to design trials to optimise treatment duration (which will often take the form of finding the shortest effective treatment duration) is, however, unclear.

The most widely used design is a non-inferiority trial;^3,4^ two key design choices are the new duration of therapy and the non-inferiority margin, that is, the maximum difference in efficacy of the new versus standard treatment duration that investigators will tolerate. If the whole confidence interval (CI) for the difference in treatment efficacy lies below this margin, non-inferiority of the shorter duration is demonstrated. However, non-inferiority trials have been often criticised;^5^ key limitations are as follows:

- The non-inferiority margin is somewhat arbitrary, typically being a multiple of 5% on the absolute difference scale. European Medicines Agency guidance^6^ recommends that the non-inferiority margin for antibiotic trials should be decided so that equivalent efficacy versus placebo can be excluded, for example, if cure rates are 80% with control and 20% without antibiotics, then the non-inferiority margin should ensure that the intervention has ≥20% cure rate. This is rarely helpful, given low cure rates for serious infections without antibiotics and high cure rates with antibiotics (also see Food and Drug Administration guidance^7^). Furthermore, at the design stage, there is often relatively little a priori information on the expected control event rate^8^ and variation even between 80% and 90% can substantially impact the sample size required to demonstrate non-inferiority on an absolute scale.
- Whether the CI should be 95% (two-sided alpha = 0.05, one-sided alpha = 0.025) or 90% (two-sided alpha = 0.10, one-sided alpha = 0.05) is still debated.
- Consequently, sample sizes for non-inferiority trials with reasonably small margins (5%) are usually very large, and they are often unsuccessful.^9^
- The shorter durations to be tested have to be chosen in advance; again, limited prior knowledge makes this choice difficult. A bad choice inevitably leads to failure of the trial or an unnecessarily long duration being adopted in clinical practice. Comparing multiple durations increases the chance of selecting sensible durations to test but requires even bigger sample sizes with the traditional design.
- There is no consensus for best analysis methods for non-inferiority trials; both intention-to-treat and per-protocol approach can lead to unreliable results. International recommendations differ;^5^ at best, leading to challenges in interpretation and, at worst, to manipulation towards the most favourable results.

An alternative approach to non-inferiority trials is therefore attractive but relatively little work has been done in this area. A recent proposal is the Desirability of Outcome Ranking/Response Adjusted for Duration of Antibiotic Risk (DOOR/RADAR) trial design.^10^ RADAR first categorises patients using a composite clinical outcome (based on benefits and harms) and then successively ranks them with respect to a DOOR, assigning higher ranks to patients with better composite outcomes and shorter antibiotic durations. Finally, the probability that a randomly selected patient will have a better DOOR if assigned to the new treatment duration is calculated. The main criticisms of DOOR/RADAR are that combining clinical outcome and treatment duration into a single composite may hide important differences in the clinical outcome alone and intrinsically assumes (rather than estimates) that shorter durations are beneficial, and hence, the clinical interpretation of the treatment effect on the composite endpoint is far from clear. Phillips et al.^11^ showed that two non-inferiority trials where shorter durations had been unequivocally demonstrated *not to be non-inferior* would have instead demonstrated non-inferiority using DOOR/RADAR.

To identify appropriate treatment durations, another possible approach is to model the duration–response curve, borrowing information from other durations when calculating treatment effect at a particular duration. This was first proposed, in a limited way, by Horsburgh et al.^12^ where, on the log-odds scale, the effect of duration on response rate was assumed to be linear (logistic regression model).

However, in general, and certainly for antibiotic treatment duration, this strong assumption is unlikely to hold. Therefore, here, we instead propose using flexible regression modelling strategies to model the duration–response curve, to provide robustness under general forms of the true duration–response curve.

## Proposals

Suppose a treatment *T* has currently recommended duration $D_{\max}$ and there is a minimum duration $D_{\min}$, we are willing to compare with $D_{\max}$, possibly because an even shorter duration is thought unlikely to be sufficiently effective. Our goal is to model the duration–response curve for response *Y* between $D_{\min}$ and $D_{\max}$. In the equations below, *Y* can be either a continuous outcome or a linear predictor of a binary outcome (representing cure). In simulations, we will assume $D_{\min}=10$ and $D_{\max}=20$.

The most appropriate design depends on the true shape of the duration–response curve; we therefore have to ensure robustness against a series of different scenarios. For example, allocating patients to only two arms, at $D_{\max}$ and $D_{\min}$ would be a very good design if the duration–response curve was linear, but a terrible design for quadratic duration–response relationships.

Therefore, instead of focusing on a single duration–response curve, we simulated data from a set of plausible duration–response curves and then evaluated several study designs across these scenarios. In particular, we explored the effect of changing: (1) total sample size *N*, (2) number and (3) position of duration arms and (4) the type of flexible regression model used.

However, to select the most accurate procedure for estimating the duration–response curve, we need to choose a measure of discrepancy between the true and estimated curves.

Lack of accuracy is often evaluated through either the integral error or the expected error. For a fixed set of chosen durations $D=(D_{1},\ldots ,D_{n})=(D_{\min},\ldots ,D_{\max})$, the expected error is defined as follows

(1) $$\mathrm{EE}=\frac{1}{n}\sum_{i=1}^{n}\Delta (f(D_{i}),\hat{f}(D_{i}))$$

where Δ represents a sensible measure of distance, for example, squared difference or absolute difference, $f(D_{i})$ represents the true response (typically probability of cure) corresponding to treatment duration $D_{i}$ and $\hat{f}(D_{i})$ represents the corresponding estimate from the fitted model. However, this sum is over the durations defining the support, for example, only over the specified durations, while we would like to evaluate the fit of the model across the whole duration range $\left[D_{\min},D_{\max}\right]$. Therefore, we instead used a type of integral error, that is, a measure of accuracy defined through an integral, instead of a sum, to characterise model accuracy over the entire domain of interest $D=[D_{\min},D_{\max}]$

(2) $$\mathrm{IE}=\int_{D\min}^{D\max}\Delta (f(D),\hat{f}(D))\mathrm{dD}$$

We chose the absolute difference as measure of distance Δ, as it has the most straightforward interpretation, namely, the area between the true and estimated duration–response curve. Henceforth, we refer to this measure as the Area Between Curves. However, this has as units probability-days which is challenging to interpret. Therefore, we divided it by $\left(D_{\max}-D_{\min}\right)$ to produce a measure on the probability scale, the scaled Area Between Curves. For a particular fitted curve, this can be interpreted as the average absolute error in the estimation of probability of cure, with respect to a uniform distribution for duration on $\left(D_{\min},D_{\max}\right)$. In some areas of the curve, the model may fit better, and in some others, it may fit worse; however, this measure provides an average across the whole duration range. We then additionally considered the maximum absolute error in $\left(D_{\min},D_{\max}\right)$ and the coverage level, defined as the proportion of the true curve included within the point-wise 95% confidence region around the estimated curve.

All these measures can only be calculated when the true underlying curve is known. They are therefore only useful for simulations to evaluate the behaviour of our proposed method.

To model the duration–response curve as flexibly as possible, we compared four different regression strategies:

1. Fractional polynomials (FP)^13,14^ of the form

(3) $$Y=\beta_{1}D^{p_{1}}+\cdots +\beta_{M}D^{p_{M}}$$

with powers $p_{1},\ldots ,p_{M}$ taken from a special set S={−2,−1,−0.5,0,0.5,1,2,3}. Usually M<3 is sufficient for a good fit; here, we fix M=2, producing 36 possible combinations.

2. Linear splines, with the simplest form, under a single knot *K*

(4) $$Y=\beta_{0}+\beta_{1}D+\beta_{2}(D-K{)}_{+}$$

where $(D-K{)}_{+}=0$ if D<K. We investigated linear splines with different numbers of knots; we present results with three or five knots. Knots are equidistant, within the duration range considered, for example, for three knots, positioned at K={12.5,15,17.5}.

3. Linear spline with non-equidistant knots: this concentrates knots for the linear splines in the first half of the duration range, where the duration–response relationship is most likely to be non-linear. We use three knots that we arbitrarily chose to position at K={11,13,15}.
4. Multivariate adaptive regression splines,^15,16^ which builds models of the form

(5) $$Y=\sum_{i=1}^{k}\beta_{i}B_{i}(D)$$

where each $B_{i}(D)$ can be (1) a constant, (2) a hinge function, that is, max(0,D−K) or max(0,K−D) or (3) a product of two hinge functions. A forward selection step, building on a greedy algorithm, is followed by a backward elimination step, to avoid over-fitting. Candidate knots *K* are all durations observed in the sample, that is, all selected duration arms.

We did not consider restricted cubic splines^17^ because preliminary work showed similar results to piece-wise linear splines; therefore, we focussed on linear splines for simplicity. Other non-linear regression methods include logistic or Gompertz growth models; however, these lose flexibility.

Other key design parameters are as follows: How many different duration arms should we allocate patients to? How should we space arms across our duration range? How many patients should we enrol? We addressed these questions in an extensive simulation study.

## Results

The eight different scenarios considered represented a wide range of possible duration–response relationships, from linear to quadratic, sigmoid curves and piecewise functions (Table 1). We simulated binary responses, representing cure of infection, from a binomial distribution with duration-specific event rates, with 1000 simulated trials for each combination of design parameters.

**Table 1.** Simulation scenarios: eight different data-generating mechanisms were investigated.

Type	Equation	Characteristics	Plot
1. Logistic growth curve	$P_{\mathrm{success}}=0.05+\frac{0.9}{1+\exp(-2D+25)}$	Increases and asymptotes early	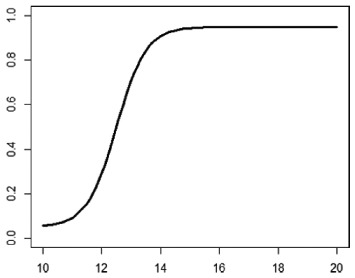
2. Gompertz curve A	$P_{success}=0.9\exp(-\exp(-0.5(D-11)))$	Small curvature	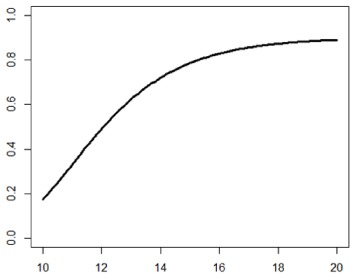
3. Gompertz curve B	$P_{success}=0.9\exp(-\exp(-(D-11)))$	Larger curvature, asymptotes more clearly	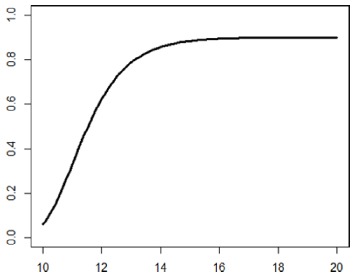
4. Gompertz curve C	$P_{success}=0.9\exp(-2\exp(-(D-9)))$	Asymptotes extremely early	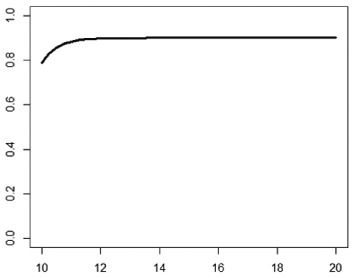
5. Linearduration–responsecurve on log-odds scale	$logit(P_{success})=0.847+0.210(D-10)$	Situation where simple logisticregression is appropriate	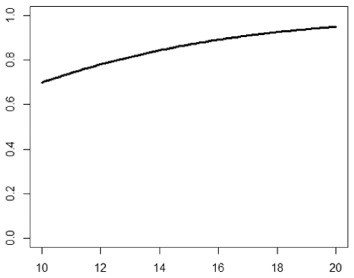
6. Quadraticduration–responsecurve, curvature > 0	$P_{success}=0.7+0.0015{\left(D-10\right)}^{2}$	First derivative increasing	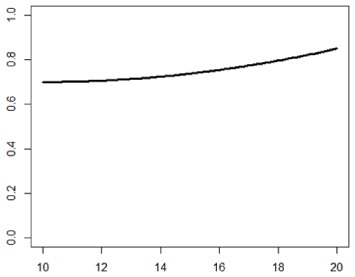
7. Quadraticduration–responsecurve, curvature < 0	$P_{success}=0.7-0.0015{\left(D-10\right)}^{2}+0.03(D-10)$	First derivative decreasing	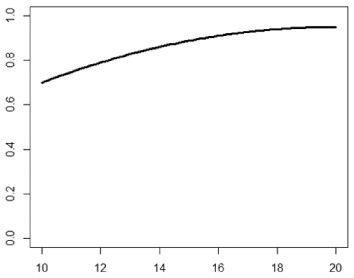
8. Piece-wise linearduration–response curve	$\begin{array}{c} P_{\mathrm{success}}=(0.5+0.15(D-10))\\ 1(D<12)+(0.8\\ +0.05(D-12))1(D<15)\\ +(0.94+0.01(D-15))1(D>15) \end{array}$	Different from linear spline logisticregression, here it is linear inthe success rate, notin the log-odds	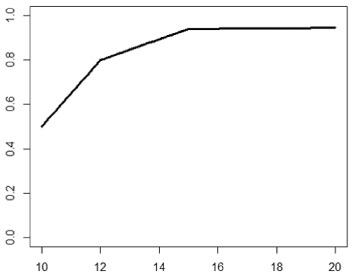

In plots, *x*-axis is treatment duration, and *y*-axis is probability of cure.

### Base-case design

We first fixed a sample size of 504 individuals randomised between seven equidistant duration arms

D={10,11.6,13.3,15,16.6,18.3,20}

We kept durations unrounded, simulating a situation where an antibiotic is administered three times a day, and therefore 11.6 means three times daily for 11 days and then twice on the last day. Simulated data were analysed with a FP logistic regression model, that is, on the log-odds scale.

In all eight scenarios, the worst fit still led to a scaled Area Between Curves below 5.3% in 95% of simulations (Table 2); that is, in each scenario, 95% of the simulated trials led to an estimated duration–response curve whose error in the estimation of the probability of cure was under 5.3%.

**Table 2.** Scaled Area Between Curves (sABC), $\max_{d}\mathrm{AE}(d)$ and coverage (%) across the eight different scenarios in the base-case design (1000 simulations of 504 patients randomised across seven arms, using FP).

	sABC					$\max_{d}\mathrm{AE}(d)$		Coverage (%)
	Min	5th percentile	Med.	95th percentile	Max	Med.	95th percentile	Mean
Scenario 1	0.019	0.022	0.032	**0.051**	0.077	0.105	0.164	61.0
Scenario 2	0.005	0.006	0.024	**0.053**	0.082	0.047	0.128	83.4
Scenario 3	0.003	0.007	0.022	**0.048**	0.079	0.055	0.123	86.8
Scenario 4	0.007	0.010	0.022	**0.039**	0.050	0.066	0.105	79.6
Scenario 5	0.000*	0.003*	0.015*	**0.030** *	0.061*	0.030*	0.078*	94.7*
Scenario 6	0.011	0.012	0.022	**0.044**	0.066	0.051	0.100	89.5
Scenario 7	0.002	0.004	0.015	**0.031**	0.056	0.033	0.082	92.9
Scenario 8	0.009	0.010	0.025	**0.041**	0.061	0.070	0.138	72.7
Overall	0.000	0.006	0.022	**0.046**	0.082	0.055	0.129	82.6

Column for the 95th percentile of scaled Area Between Curves is in bold, to show how scaled Area Between Curves is smaller, or close to, 5% in all scenarios and overall across all 8000 simulations. Asterisks next to Scenario 5 results indicate that this is the only scenario where the data-generating mechanism is actually a particular case of fractional polynomial on the log-odds scale and therefore performs optimally. sABC is the scaled Area Between Curves as defined in the proposals section, while $\max_{d}\mathrm{AE}(d)$ indicates the maximum absolute error for a single duration $d\in (D_{\min},D_{\max})$ and coverage (%) is defined as the percentage of the true underlying curve included within the point-wise 95% confidence region around the estimated curve.

**Figure 1.**
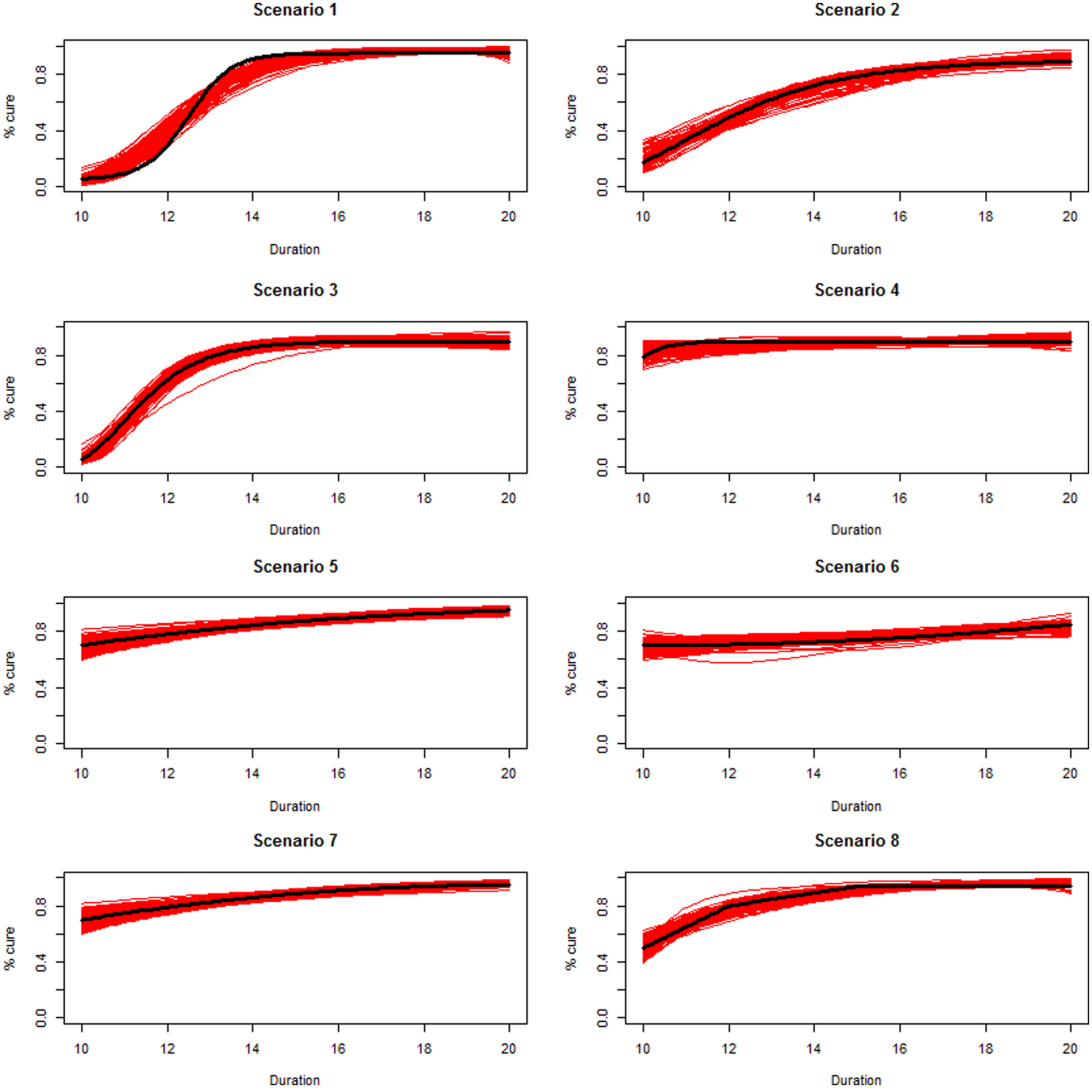
Prediction curves (red) of a random selection of 100 simulations against the true data-generating curve (black) for all the eight scenarios under the base-case configuration. The base-case scenario assumes a sample size of 504 patients, randomised to seven equidistant arms, and fits a fractional polynomial model to estimate the duration–response curve.

Scenarios 1, 2 and 3 had the poorest performance. Figure 1 shows the fitted prediction curves for a random sample of 100 simulations (red) against the true data-generating curve (black). In Scenario 1, FP had difficulty in capturing satisfactorily the substantial change in curvature around days 12 and 14, tending to underestimate curvature at these time points.

Best performances were obtained with Scenario 5, where the true duration–response curve is linear on the log-odds scale, which is exactly an FP model, with a single parameter for the term with power p=1. Similar results were obtained for Scenario 7.

The maximum scaled Area Between Curves was smaller than 10% in all scenarios, meaning that even the simulation leading to the worst fitted prediction curve led to a total bias under 10% in all scenarios.

The median of the maximum absolute error was 5.5% across all simulations, and <7% except for Scenario 1, meaning that, irrespective of the real data-generating mechanism, in half of the simulations even the single design point corresponding to the worse fit had an absolute error below 5.5%. When considering the 95th percentile of the same measure, this was just below 13% overall. Figure 5 (online supplementary material) shows that durations corresponding to the worst absolute error tended to be in the first part of the curves, where treatment was less effective.

Mean coverage was 95% only for Scenario 5, where the analysis model was correctly specified; however, most scenarios had coverage greater than 80% and Figure 6 (online supplementary material) shows that even the 100 simulations leading to the worst coverages approximated the true duration–response curve quite well for a wide variety of scenarios, similar to the randomly selected predictions in Figure 1.

Next, we investigated the sensitivity of these results to the choice of design parameters and analysis methods.

### Different flexible regression strategies

We re-analysed the same simulated data in Table 2 using either FP, linear spline with 3 or 5 equidistant knots, linear spline with knots concentrated in the first half of the curve and multivariate adaptive regression splines. Only Scenario 5 is the true model for both data generation and analysis.

**Figure 2.**
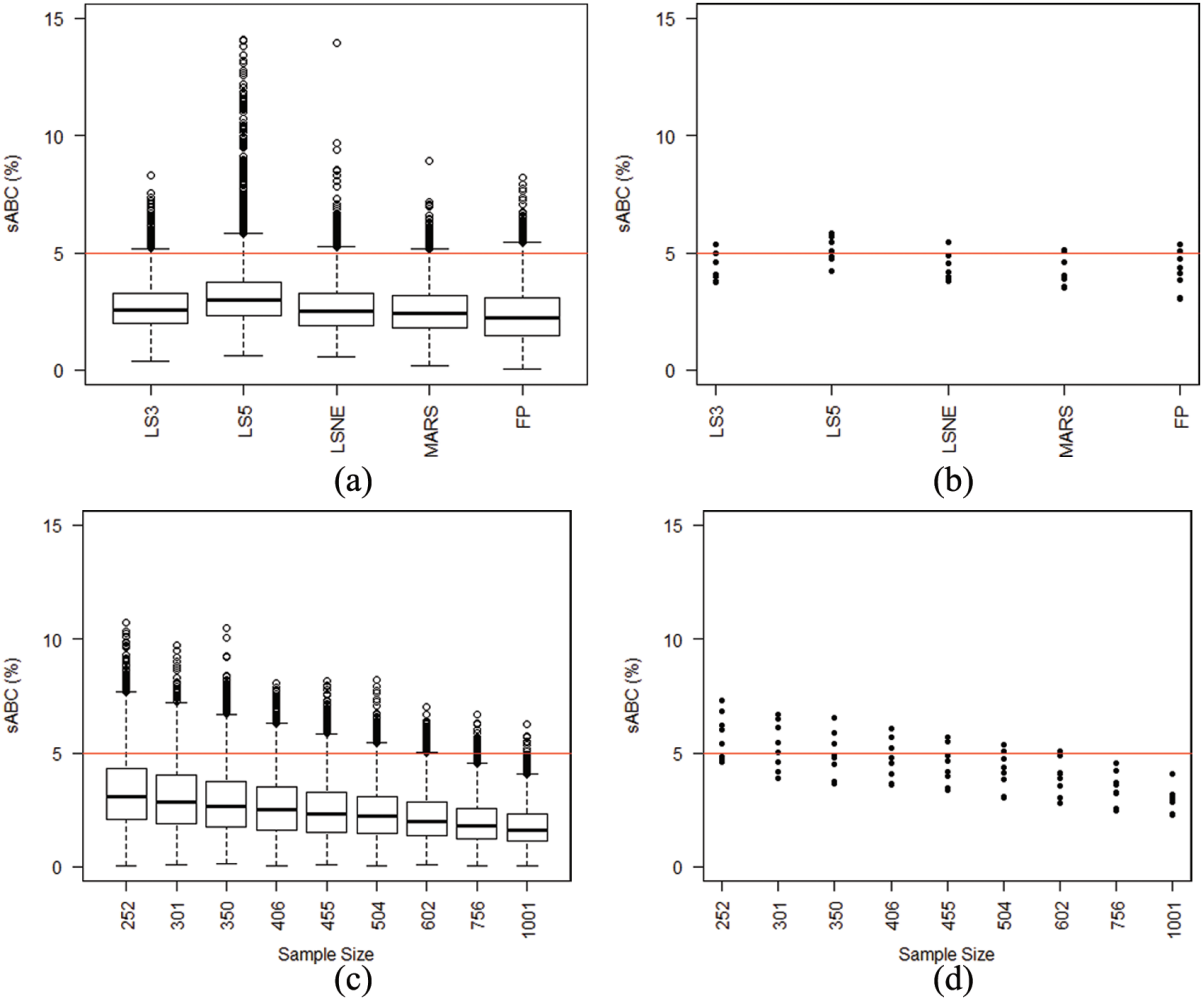
Comparison of results of trial simulations from the eight scenarios varying either (1) the flexible regression method used (LS3, LS5, LSNE, MARS, FP), with total sample size of 504 patients (panels (a) and (b)), or (2) the total sample size between 250 and 1000 patients, using FP (panels (c) and (d)). Patients are divided into seven equidistant duration arms. The red horizontal line indicates 5% scaled Area Between Curves (sABC). In the left panels, we show the box plots of the whole simulation results, while in the right panels we compare 95th percentiles from the eight scenarios. LS3-5: Linear Spline with 3–5 knots; LSNE: linear spline with non-equidistant knots; MARS: multivariable adaptive regression splines; FP: fractional polynomials. (a) Comparison of flexible regression methods: 8000 simulations. (b) Comparison of flexible regression methods: 95th percentiles. (c) Sensitivity to sample size: 8000 simulations. (d) Sensitivity to sample size: 95th percentiles.

For all methods, scaled areas for the fitted prediction curves were fairly similar (Figure 2(a) and (b)). The only method with slightly inferior performance was five-knot linear spline. FP had the smallest mean-scaled Area Between Curves across the eight scenarios, but marginally higher variability between different scenarios. FP were best in terms of smallest maximum absolute error, while splines better behaved in terms of coverage (Figure 7, online supplementary material).

**Figure 3.**
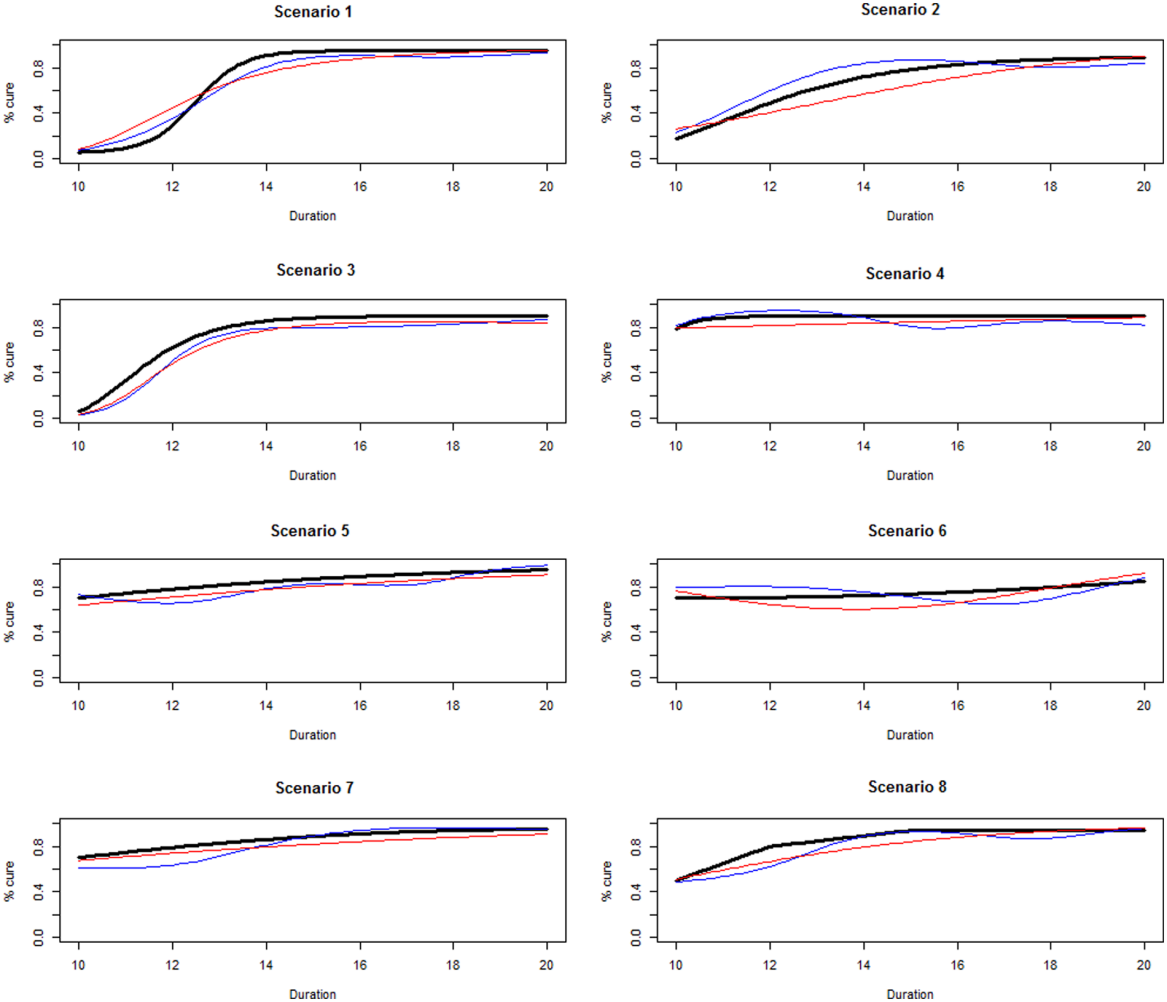
Prediction curves leading to the largest scaled Area Between Curves for each of the eight scenarios with the base-case design, analysing data either with three-knot linear spline (blue) or fractional polynomials (red).

Finally, FP had an advantage in terms of monotonicity, as shown in Figure 3, comparing prediction curves for the simulated data set with the worst fit (largest scaled Area Between Curves), across the eight scenarios, with FP (red) or three-knot linear spline (blue). Spline-based methods led to undulating functions, particularly in Scenarios 4, 5, 6 and 8, while FP prediction curves were smoother and, at least approximately, monotonously increasing, the only exception being the worst fit from Scenario 6. Spline-based methods led to even worse prediction curves in other scenarios, particularly with smaller sample sizes (e.g. 250 patients) and with poor knot positioning relative to arms, for example, two adjacent knots with no arm in between.

### Total sample size

One motivation for this study was large sample sizes often required for non-inferiority trials. We therefore investigated the sensitivity of simulation results to total sample sizes varying across N=(252,301,350,406,455,504,602,756,1001) (each divisible by seven arms).

As expected, increasing total sample size reduced the scaled Area Between Curves (second row of Figure 2). With N≥350, in more than half the scenarios, the 95th percentile for scaled Area Between Curves was under 5%, and in all scenarios for N≥750. Therefore, above this threshold, whatever the true data-generating curve, in at least 95% of simulated trials, we estimated a duration–response curve whose error was lower than 5%.

Figure 2 and Table 2 suggest that our base-case scenario sample size of 504 might be a reasonable compromise, guaranteeing good estimation of the duration–response curve without requiring too many patients.

### Number of duration arms

**Figure 4.**
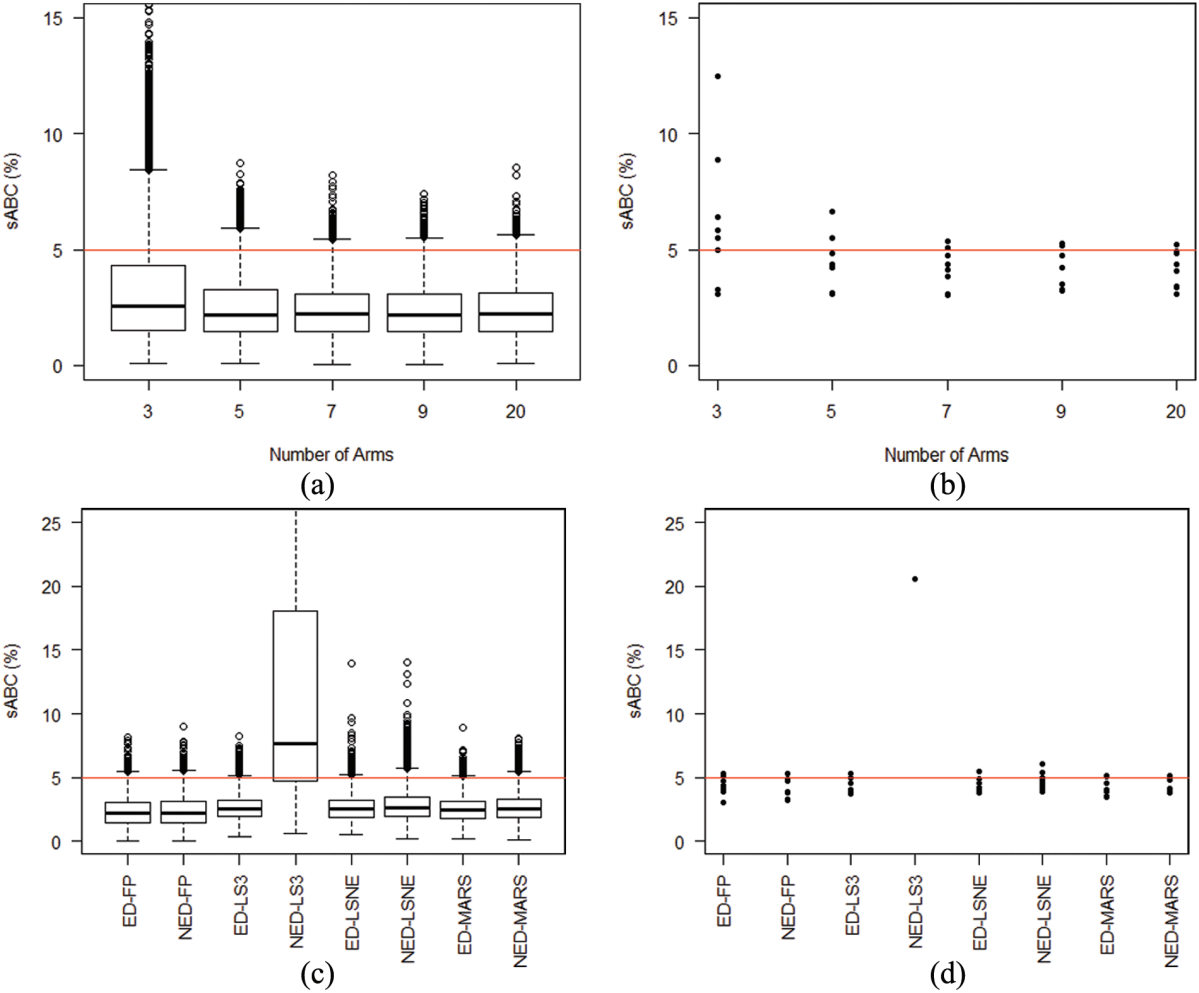
Comparison of results of trial simulations from the eight scenarios either varying the number of equidistant arms (panels (a) and (b)) between 3 and 20, using fractional polynomials (FP), or using different designs, equidistant (ED) or not equidistant (NED), comparing four different regression methods (panels (c) and (d)). The total sample size is always 504 patients. The red horizontal line indicates 5% scaled Area Between Curves. In the left panels, we show the box plots of the whole simulation results, while in the right panels, we compare 95th percentiles from the eight scenarios. In panel (d), there is only one point for NED-LS3, since only in one scenario the 95th percentile for scaled Area Between Curves was smaller than 0.25. LS3: linear spline with three knots; LSNE: linear spline with non-equidistant knots; MARS: multivariable adaptive regression splines; FP: fractional polynomials. (a) Sensitivity to number of arms: 8000 simulations. (b) Sensitivity to number of arms: 95th percentile. (c) Sensitivity to placement of arms: 8000 simulations. (d) Sensitivity to placement of arms: 95th percentile.

Figure 4(a) and (b) compares results from allocating the same number of patients (~504) to 3, 5, 9 or 20 arms, rather than the base case of 7 arms.

The three-arm design was clearly inferior and generally led to worse scaled Area Between Curves. All other designs had similar performance, and particularly distributions from 7, 9 and 20 arms appeared virtually identical, suggesting that, compared to a base-case of 7 duration arms, there is little gain from adding additional arms while keeping sample size fixed.

### Position of arms

Finally, we investigated the sensitivity of results to the position, rather than the number, of duration arms, by comparing the following:

- The standard seven equidistant arms design;
- A ‘not-equidistant’ arms design, with five arms condensed in the first part of the curve, that is, A={10,11,13,15,20}.

As for the linear spline regression model, the motivation for this choice is that the early part of the curve is where the linearity assumption is least likely to hold.

With FP, results were similar with both designs (Figure 4(c) and (d)). This is mainly because the eight scenarios have at most modest departure from linearity in the second half of the curve.

The three-knot spline regression performed particularly poorly with the ‘not-equidistant’ design, highlighting the issue of knot choice with spline-based methods. If knots are chosen inappropriately, for example, two adjacent knots with no arms in between, as here, then results may be highly variable. While obvious in this case, similar issues with inappropriate knot positioning might be less trivial to identify in other situations. In contrast, FP regression is standardised and does not require users to make additional choices.

## Extensions

Having demonstrated promising performance of our proposed method, several issues remain. The first is accounting for uncertainty. Point-wise confidence bands around the estimated curve can be calculated from the FP regression and were used here to estimate coverage levels. These intervals were generally quite narrow, the mean width around the estimated cure rate ranging between 7% and 10% in the base-case scenarios. However, these do not account for model selection uncertainty.^18^ Broadly, since we use the same set of data that we want to analyse to select the final model of interest, the usual standard error estimates from the model tend to be too small. Therefore, a measure of precision of our estimated duration–response curve would require methods, such as bootstrap model averaging.^19–21^

The second issue is how the estimated duration–response curve might be used. Possible approaches that decision makers could take given the estimated curve include the following:

1. Estimating the minimum duration that achieves a certain fixed acceptable cure rate (e.g. >80%) analogous to a cost-effectiveness acceptability curve,^22^ together with a CI. We then would be 95% confident that the upper bound would give us a cure rate greater or equal to 80%.
2. Alternatively, if we did not know the true control success rate, estimating the duration leading to a certain acceptable loss in efficiency compared to the maximum duration tested, for example, 10%.
3. The information gathered from the estimated curve could also be combined with other information about toxicity or cost in a decision analytic framework. This could be particularly appealing in the example of hepatitis C, where cost is quantifiable, but would be more complex in the antibiotic example, where resistance is more complex.

## Discussion

We have proposed a new design for randomised trials to find effective shorter durations of treatment, for example, antibiotics, broadening a previous suggestion.^12^ The underpinning concept is, instead of directly comparing a limited and arbitrarily chosen number of particular durations, to model the whole duration–response curve across a pre-specified range of durations, in order to maximise the information gained about the effect of shorter or longer regimens. The resulting estimate of the dose–response curve could then be used in a variety of clinically meaningful ways.

Because of lack of information on the true shape of this duration–response curve, we used flexible modelling strategies, to protect against parametric model misspecification. We compared four different strategies, three based on splines and one on FP, concluding that, although spline-based methods can potentially better estimate locally the duration associated with a particular cure rate, FP are better at providing a reasonable curve describing the evolution of the cure rate over treatment duration. Binder et al.^23^ conducted a vast simulation study comparing FP and spline-based methods, broadly concluding that with large data sets, the two methods lead to similar results, while in medium-sized data sets FP outperform spline-based methods on several criteria. They also noted that a major advantage of FP is the simplicity of implementation in standard software packages, compared to the absence of recommendations regarding appropriate spline-based methods, matching our conclusions.

While we could have used FP with more than two polynomials, we focussed on two to reduce the number of parameters, having only a small number of duration arms in our setting. Similarly, we focussed on the standard set of possible powers, but higher powers could be considered, if thought likely to improve fit. FP and multivariate adaptive regression splines’ implementation in standard software packages does not allow restriction to monotonously increasing functions; since it is reasonable to assume monotonicity of the duration–response curve, this could be explored in future.

Regarding design parameters, a modest number of equidistant arms, for example, 7, appeared sufficient to give robust results, that is, the resulting prediction curve from the fit of the model was reasonably close to the true underlying duration–response curve and can therefore provide sufficient information for clinicians about the effect of duration on treatment response. The ‘not-equidistant’ design provided similar results with only five arms (but the same number of patients); however, such a design might be less robust to other shapes of the duration–response curve, for example, if the curve was far from linear even in the second part of the duration range investigated.

When multi-arm multi-stage designs were first mooted, multiple arms were often raised as a theoretical barrier to recruitment, but subsequent practice has demonstrated that, if anything, these trials are more acceptable to patients, since they ably demonstrate equipoise between a substantial number of treatment options.^24^

One legitimate criticism of non-inferiority trials is the arbitrary nature of the non-inferiority margin; in our framework, since $D_{\max}$ represents the currently recommended treatment duration, the only arbitrary choice is that of the minimum duration to be considered, $D_{\min}$. This choice certainly has a much smaller impact on the trial results than the choice of a non-inferiority margin, but nevertheless it is still extremely important to choose this carefully. Since we lack any information about the true shape of the duration–response curve below the currently recommended duration, $D_{\max}$, a multi-stage adaptive design could be used to change the position of $D_{\min}$ if results after a first stage clearly showed this to be too long (i.e. the shortest duration still leading to high efficacy) or too short (i.e. duration extremely ineffective, which might be considered unethical to keep randomising patients to).

Here, we have considered models where the only covariate was treatment duration; however, it would be interesting to investigate the effect of incorporating additional covariate data, such as age and sex. This could be done as a main effect, for example, to adjust the minimum duration needed to achieve a threshold cure rate according to other characteristics affecting cure; alternatively, this could be done as an interaction, providing a different duration–response curve for specified subgroups, for example, males versus females. Either would allow stratified or personalised medicine, allowing clinicians to prescribe different durations according to key patient characteristics.

The underpinning motivation for this article was a phase IV trial design to identify minimal effective antibiotic treatment duration, and the design could be applied to other similar situations. However, an evaluation of the inferential properties of the methodology is key before recommending it in these late-phase settings; in particular, preservation of type I error rate is fundamental, as these are treatments that are known to be effective, and recommending an insufficiently long duration could potentially have serious public health consequences. Once this is done, examples of applications may include phase III trials in tuberculosis, where shorter treatment durations could improve adherence compared to standard-of-care control duration, or phase IV trials in hepatitis C where current treatment regimens achieve cure in >95% of patients but are extremely costly. Similar approaches could be applied to dose-intensity of chemotherapy regimens.

The problem addressed here has similarities with that of finding the optimal treatment dose in early-phase clinical trials. There is a vast literature on methods for modelling dose–response relationship to find optimal treatment doses.^25,26^ However, there are important differences making it difficult to use those methods in our situation. The sample sizes required are much smaller in dose–response studies because the guiding principle is to start with a low dose and to increase it, avoiding exposing too many patients to excessive, and thus unsafe, doses. This is usually done before the drug has actually been tested in phase II–III trials. The power of these methods to identify the correct minimum effective dose is therefore often quite low.^27^ With larger sample sizes, methods like the Continual Reassessment Method become infeasible, most of all in the example of tuberculosis where treatment may last several months. Furthermore, in early-stage trials, the focus is often on pharmacokinetics, and the specific forms of the dose–response curves used usually derive from the underlying pharmacokinetic models for drug absorption into the bloodstream.

In conclusion, our proposed new paradigm for clinical trials to optimise treatment duration has the potential to revolutionise the design of trials where reducing treatment duration is our goal, for example in the fight against antimicrobial resistance. Our approach moves away from multiple inefficient trials of arbitrary antibiotic durations that may all be suboptimal. We have shown how certain design parameters may affect the fit of a flexible regression strategy to model the duration–response curve. Randomising approximately 500 patients between a moderate number of equidistant arms (5–7) is sufficient under a range of different possible scenarios to give a good fit and describe the duration–response curve well. Further work on how to use this estimated curve to draw inference, controlling power and type I error rate, will follow.
